# Nationwide implementation of minimally invasive liver surgery: population-based analysis

**DOI:** 10.1093/bjsopen/zraf164

**Published:** 2026-01-21

**Authors:** Emil Östrand, Jenny Rystedt, Bobby Tingstedt, Bodil Andersson

**Affiliations:** Department of Clinical Sciences Lund, Surgery, Lund University, Lund, Sweden; Department of Surgery, Skåne University Hospital, Lund, Sweden; Department of Clinical Sciences Lund, Surgery, Lund University, Lund, Sweden; Department of Surgery, Skåne University Hospital, Lund, Sweden; Department of Clinical Sciences Lund, Surgery, Lund University, Lund, Sweden; Department of Surgery, Skåne University Hospital, Lund, Sweden; Department of Clinical Sciences Lund, Surgery, Lund University, Lund, Sweden; Department of Surgery, Skåne University Hospital, Lund, Sweden

## Abstract

**Background:**

Previous studies of minimally invasive liver surgery described results and experiences in high-volume centres and early adopters, but data on national levels are lacking. This study evaluated the implementation and outcomes of minimally invasive liver surgery in Sweden over a 15-year period, with a focus on colorectal liver metastases.

**Methods:**

Data from patients undergoing liver surgery between 2009 and 2023 were obtained from the Swedish National Quality Registry for Liver, Gallbladder and Bile Duct Cancer, and evaluated in time intervals. Propensity score matching analysis was used to compare outcomes between open and minimally invasive liver surgery for colorectal liver metastases.

**Results:**

A total of 9977 procedures were included in the study, of which 1490 (14.9%) were minimally invasive. Minimally invasive liver surgery was used increasingly over time, and had better short-term outcomes than open liver operations, including less blood loss (median 200 (interquartile range 50–400) *versus* 500 (250–1000) ml; *P* < 0.001), fewer major complications (127 (9.3%) *versus* 1697 (21.9%); *P* < 0.001), and a lower 30-day mortality rate (6 patients (0.4%) *versus* 107 (1.3%); *P* = 0.004). Use of robotically assisted liver surgery increased over time and it constituted 311 minimally invasive liver procedures (38.4%) in the late time period. Propensity score matching analysis for patients with colorectal liver metastases showed reduced blood loss with minimally invasive liver surgery (*P* < 0.001), a similar rate of radical resections, and similar overall survival.

**Conclusion:**

The study demonstrated safe nationwide implementation of minimally invasive liver surgery. Use of the minimally invasive approach increased over time, including a rapid rise for robotically assisted procedures in the later period. Minimally invasive liver surgery maintained or improved favourable short-term outcomes without adverse effects on morbidity, mortality or long-term survival after surgery for colorectal liver metastases.

## Teaser

The study analysed the implementation of minimally invasive liver surgery (MILS) in Sweden over 15 years. The rate of MILS increased over time, including a fast rise in robotically assisted liver surgery in the later period. MILS maintained or improved favourable short-term outcomes without adverse effects on morbidity, mortality or long-term survival for patients with colorectal liver metastases, in the full cohort and a propensity score-matched cohort.

## Introduction

Laparoscopic liver surgery was first reported more than 30 years ago^1^, and has since become a standard practice in hepatobiliary centres^2^. Several studies^2–6^ have shown similar oncological outcomes after minimally invasive liver surgery (MILS) compared with open liver surgery (OLS), and demonstrated advantages in short-term outcomes, such as less bleeding, less pain, shorter hospital stay, and lower complication rates. Initial concerns regarding technical difficulties, limitations, risk of substandard resection margins, and worse long-term outcomes in MILS have been acknowledged and recommendations for safe adoption of MILS have been made^2,7–9^. The technical difficulty of laparoscopic liver surgery can be assessed by means of difficulty scoring systems, such as the Institut Mutualiste Montsouris (IMM) classification, and appropriately experienced laparoscopic surgeons are recommended to perform the more difficult resections, accordingly^2,10–12^. Robotically assisted liver surgery (RALS) has been introduced as an alternative with improved freedom of movement, three-dimensional visualization, and a shorter learning curve, and studies^13–18^ have shown short-term outcomes similar to those of laparoscopic liver surgery. Long-term oncological outcomes for RALS have been studied less, but results are comparable to those of laparoscopy^19,20^.

Most earlier publications on MILS described implementation by early adopters and in high-volume centres, but data are limited at a population-based national level. Regarding gastrointestinal resectional surgery, including liver surgery, open surgery seems to have remained the norm in Sweden for a long time^21,22^. In 2008, the Swedish National Quality Registry for Liver, Gallbladder and Bile Duct Cancer (SweLiv) was started for liver, gallbladder, and bile duct cancer, but also all liver operations regardless of diagnosis and covering the entire population. This provides a unique opportunity to observe and evaluate the implementation of new procedures or techniques^22^.

The aim of the present study was to describe and evaluate the implementation of MILS in a national cohort and to compare results between MILS and OLS, with a special focus on colorectal liver metastases (CRLMs).

## Methods

### Patients and data collection

This population-based observational study was conducted according to the STROBE guidelines for cohort studies^23^. The data used in the study were retrieved from SweLiv. The registry has coverage rates of > 95% compared with the compulsory Swedish cancer registry, and patients undergoing liver surgery in Sweden are registered prospectively^22^. Data were retrieved on 17 October 2023. All consecutive patients who underwent resection between 1 January 2009 and 16 October 2023 were included. Exclusion criteria were: transplantation surgery, bile duct or gallbladder surgery alone without liver resection, hyperthermic liver perfusion surgery, portal vein ligation or embolization only, ablations only, or other non-hepatic procedures. Duplicates were excluded. Ethical approval for this study was obtained from the Swedish Ethical Review Authority (2023–04042-01). As patients were already participating in the registry, no additional informed consent was required.

### Definitions

Until 2019, operative procedure codes were used to define whether surgical access was minimally invasive or open, to identify RALS, and whether surgery was converted to an open approach. From 2020 the registry had a new variable regarding the surgical approach. Operations converted to open surgery were analysed by intention to treat and thus considered as MILS in the analyses. The Clavien–Dindo classification was used to compare complications^24^. Major complications were defined as those with a Clavien–Dindo grade ≥ IIIa. As complications were registered differently in SweLiv during the study period, they were combined into a single compound variable. The size of the largest tumour and the number of tumours were retrieved from the pathology section of the registry or, if missing, from preoperative assessments. Major hepatic resections were defined as anatomical resections of three or more connected Couinaud segments. Extrahepatic disease was defined as known extrahepatic metastasis at the time of surgery. Technical difficulty was graded retrospectively, according to the IMM score (*Appendix S1*)^10–12^. Radical resections were defined as those with a margin of ≥ 1 mm for CRLM, according to pathology reports, and regarded as missing data if the margin status was registered as indeterminate. Phases of the learning curve were defined using the cumulative number of procedures performed per institution, as competency (34 procedures), proficiency (50), and mastery (58) phases^25^. Performance status (PS) was classified according to the Eastern Cooperative Oncology Group^26^ . Overall survival (OS) was calculated from the date of liver surgery until the date of death from any cause. Length of hospital stay (LOS) was calculated from the date of operation until discharge to home.

To evaluate development over time, operations were analysed by comparing variables in three time periods: early (2009–2013), middle (2014–2018), and late (2019–2023).

### Statistical analysis

Data are presented as median (interquartile range, i.q.r.), or numbers with percentages, as appropriate. The χ^2^ test was used for analysis of categorical data, and the Mann–Whitney *U* or Kruskal–Wallis test for continuous data, except for the matched cohort when the Wilcoxon signed-rank test was used for continuous data, and McNemar’s χ^2^ test for categorical data to account for the within-match dependence. In the largest subgroup of patients, those who had resection of CRLM, subgroup analyses were performed for patients undergoing resection for the first time. To reduce selection bias and achieve a balanced comparison, propensity score matching (PSM) was employed in the CRLM cohort. PSM was carried out as described by Austin^27^ using nearest-neighbour matching (1 : 1) with a caliper width of 0.01. In the matched cohort, standardized mean differences of ≤ 0.10 were accepted. Variables included in the propensity score construction were age, sex, American Society of Anesthesiologists (ASA) grade, number of metastases ≥ 3, size of largest metastasis ≥ 3 cm, IMM score, simultaneous ablations, neoadjuvant chemotherapy, bilobar resections, major hepatectomies, anatomical resections, and extrahepatic metastases. OS was estimated from Kaplan–Meier curves in the CRLM cohort, and treatment groups were compared using log rank tests; a Cox regression with shared frailty was used in the matched cohort to account for within-match dependence. For internal validity, a Cox regression analysis adjusted for all variables included in the matching was undertaken in the full cohort. In survival analyses, only data from the first liver surgery were considered. For two-stage resections, the first date of surgery was used for survival analysis. Data on LOS were analysed from 2020 and were compared in the matched cohort only for relevance. Subgroup analyses were also performed for converted procedures, and for laparoscopic procedures compared with RALS.

Results were considered statistically significant at *P* < 0.050. Statistical analyses were undertaken using SPSS^®^ version 29.0.0.0 (IBM, Armonk, NY, USA) and R version 4.3.3 (R Foundation for Statistical Computing, Vienna, Austria).

## Results

**Fig. 1:**
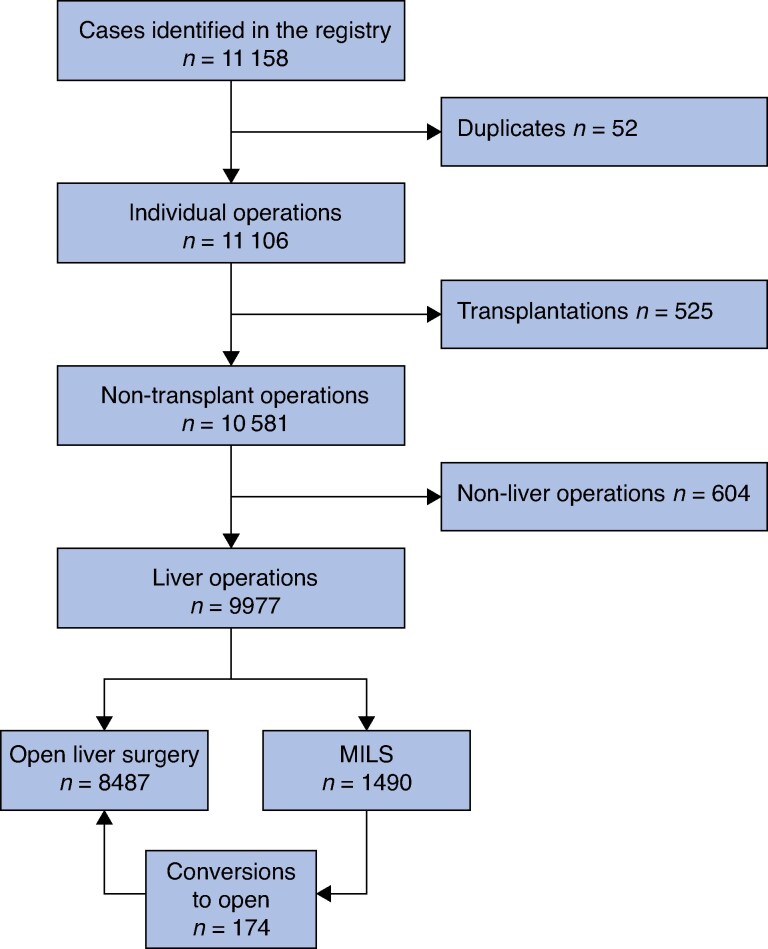
Study flow chart Patients were excluded if they underwent transplantation surgery, or non-liver resections. MILS, minimally invasive liver surgery.

A total of 9977 liver operations in 9019 patients were included, with 1490 (14.9%) starting minimally invasively, of which 174 (11.7%) were converted to OLS (*Fig. 1*). Compared with OLS, women and patients with lower PS were more frequently selected for MILS (*P* = 0.002 and *P* = 0.011 respectively), whereas age and ASA grades had a similar distribution in the two approaches (*Table 1*). Major hepatectomies, bilobar resections, anatomical resections, simultaneous ablations, and synchronous other surgery were more common in the OLS cohort (*P* < 0.001). Intraoperative blood loss was less (median 200 (i.q.r. 50–400) *versus* 500 (250–1000) ml (*P* < 0.001), the complication rate was reduced, and more patients had complete recovery of PS within 30 days (820 (75.5%) *versus* 3567 (65.1%); *P* < 0.001) in the MILS compared with the OLS cohort.

**Table 1:** Characteristics of OLS and MILS cohorts

	Missing data	OLS (*n* = 8487)	MILS (*n* = 1490)	Total (*n* = 9977)	P*
Age (years), median (i.q.r.)	2 (0.0)	66 (58–73)	66 (56–74)	66 (58–73)	0.721†
**Sex**	0 (0%)				0.002
Male		4898 (57.7%)	796 (53.4%)	5694 (57.1%)	
Female		3589 (42.3%)	694 (46.6%)	4283 (42.9%)	
ASA grade ≥ III	314 (3.1%)	2265 (27.5%)	401 (27.9%)	2666 (27.6%)	0.785
Preoperative PS, ECOG ≥ 2	1642 (16.5%)	541 (7.7%)	78 (5.8%)	919 (7.4%)	0.011
**Indication for liver surgery**	4 (0.0%)				<0.001
Primary liver malignancy		1619 (19.1%)	282 (18.9%)	1901 (19.1%)	
Gallbladder cancer		548 (6.5%)	66 (4.4%)	614 (6.2%)	
Benign or uncertain		938 (11.1%)	413 (27.7%)	1351 (13.5%)	
Liver metastases		5379 (63.4%)	727 (48.9%)	6107 (61.2%)	
Tumour size ≥ 3 cm	1188 (11.9%)	3662 (48.9%)	527 (40.8%)	4189 (47.7%)	<0.001
> 3 tumours	485 (4.9%)	1237 (15.4%)	24 (1.7%)	1261 (13.3%)	<0.001
**Type of resection**	666 (6.7%)				
Anatomical		3321 (40.3%)	419 (30.4%)	3740 (38.9%)	<0.001
Non-anatomical		3077 (37.4%)	855 (62.0%)	3827 (40.9%)	
Both		1837 (22.3%)	106 (7.7%)	1943 (20.2%)	
Bilobar resection	496 (5.0%)	3530 (43.6%)	266 (19.1%)	3796 (40.0%)	<0.001
Simultaneous ablation	0 (0%)	784 (9.2%)	44 (3.0%)	828 (8.3%)	<0.001
Simultaneous other surgery	2243 (22.5%)	712 (11.1%)	77 (5.9%)	789 (10.2%)	<0.001
Major hepatectomy	210 (2.1%)	2961 (35.7%)	47 (3.2%)	3008 (30.8%)	<0.001
**Difficulty score**	404 (4.0%)				<0.001
IMM1		2648 (32.3%)	881 (63.9%)	3529 (36.97%)	
IMM2		1078 (13.2%)	133 (9.7%)	1211 (12.7%)	
IMM3		4469 (54.5%)	364 (26.4%)	4833 (50.5%)	
Blood loss (ml), median (i.q.r.)	375 (3.8)	500 (250–1000)	200 (50–400)	400 (200–900)	<0.001†
**Complications**	847 (8.5%)				<0.001
None		4611 (59.4%)	1044 (76.1%)	5655 (61.9%)	
Minor		1451 (18.7%)	200 (14.6%)	1651 (18.1%)	
Major		1697 (21.9%)	127 (9.3%)	1824 (20.0%)	
30-day mortality	10 (0.1%)	107 (1.3%)	6 (0.4%)	113 (1.1%)	0.004
90-day mortality	11 (0.1%)	233 (2.7%)	21 (1.4%)	254 (2.5%)	0.003
PS fully recovered at 30 days	3413 (34.2%)	3567 (65.1%)	820 (75.5%)	4387 (66.8%)	<0.001

Values are *n* (%) unless otherwise stated. Cohort includes all patients regardless of diagnosis. OLS, open liver surgery; MILS, minimally invasive liver surgery; i.q.r., interquartile range; ASA, American Society of Anesthesiologists; PS, performance status according to Eastern Cooperative Oncology Group (ECOG); IMM, Institut Mutualiste Montsouris. *χ^2^ test, except †Mann–Whitney *U* test.

**Fig. 2:**
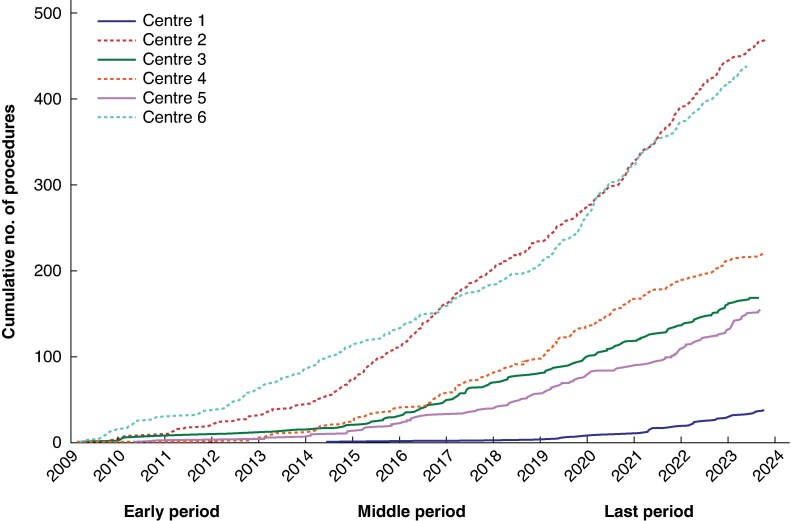
Learning curves for Swedish hepatopancreatobiliary centres Cumulative number of minimally invasive liver operations performed in Swedish hepatopancreatobiliary centres. Phases of the learning curve were defined using the cumulative number of procedures per institution, as competency (34), proficiency (50), and mastery (58) phase.

Over the early, middle, and late periods, 164 (6.5%), 516 (13.9%), and 810 (21.8%) procedures were MILS. The proportion of MILS increased over time for all surgical indications, the largest proportion being observed when the indication for surgery was benign or uncertain malignancy, increasing from 36 procedures (13.2%) in the early time period to 244 (41.2%) in the most recent period (*Table S1*). The rate of bilobar resections increased over time in the MILS cohort (15 (9.6%) *versus* 185 (24.4%); *P* < 0.001) (*Table 2*). The rate of resections with a high level of difficulty (IMM3) decreased (63 (40.4%) to 172 (22.6%); *P* < 0.001). IMM3 resections decreased over time for OLS as well (from 1362 (58.1%) in early period to 1709 (54.4%) and 1396 (51.6%) in middle and later periods respectively; *P* < 0.001), and so did major resections (977 (33.0%) *versus* 1092 (34.3) and 889 (32.5%)). The rate of complications after MILS was higher in the middle period (*P* = 0.031). The use of RALS increased over time, from 8 (4.9%) and 7 (1.4%) in the early and middle periods to 311 (38.4%) of all MILS in the late period. The rate of conversion from MILS to open surgery increased over time, from 1 (0.6%) in the early period, to 31 (6.0%) in the middle period, and 142 (17.5%) in the late period (*P* < 0.001). Converted procedures were more often complex operations, with more major resections (*P* < 0.001), more bilobar resections (*P* < 0.001), and higher difficulty scores (IMM2–3 80 *versus* 417; *P* < 0.001), resulting in increased blood loss (*P* < 0.001), worse complications, and a lower PS recovery rate in 30 days (*P* < 0.001) (*Table 3*). One of the six Swedish hepatopancreatobiliary centres did not perform any MILS in the early period, and the various centres reached the cumulative number of procedures defining the competency, proficiency, and mastery phases of the learning curve in different time periods (*Fig. 2*). The rate of MILS increased over time in all centres, with a distribution in the late period from 798 (10.7%) to 231 (36.3%).

**Table 2:** Data for MILS cohort over time

	Missing data	Early period (2009–2013) (*n* = 164)	Middle period (2014–2018) (*n* = 516)	Late period (2019–2023 (*n* = 810)	Total (*n* = 1490)	P*
Age (years), median (i.q.r.)	0 (0)	66 (58–73)	65 (56–72)	66.5 (56–75)	66 (56–74)	0.023†
**Sex**	0 (0%)					0.662
Male		93 (56.7%)	275 (53.3%)	428 (52.8%)	796 (53.4%)	
Female		71 (43.3%)	241 (46.7%)	382 (47.2%)	694 (46.6%)	
ASA grade ≥ 3	52 (3.5%)	35 (21.5%)	109 (21.5%)	257 (33.5%)	401 (27.9%)	<0.001
Preoperative PS, ECOG ≥ 2	136 (9.1%)	5 (5.3%)	25 (4.9%)	48 (4.6%)	78 (5.8%)	0.516
Tumour size ≥ 3 cm	197 (13.2%)	74 (47.1%)	185 (38.1%)	268 (41.2%)	527 (40.8%)	0.131
> 3 tumours	47 (3.2%)	3 (1.9%)	10 (2.0%)	11 (1.4%)	24 (1.7%)	0.722
**Type of resection**	110 (7.4%)					<0.001
Anatomical		60 (38.5%)	152 (32.8%)	207 (27.2%)	419 (30.4%)	
Non-anatomical		76 (48.7%)	282 (60.9%)	497 (65.3%)	855 (62.0%)	
Both		20 (12.8%)	29 (6.3%)	57 (7.5%)	106 (7.7%)	
Bilobar resections	98 (6.6%)	15 (9.6%)	66 (13.8%)	185 (24.4%)	266 (19.1%)	<0.001
Simultaneous ablation	0 (0%)	3 (1.8%)	12 (2.3%)	29 (3.6%)	44 (3.0%)	0.280
Simultaneous other surgery	178 (12%)	1 (3.4%)	33 (6.4%)	43 (5.6%)	77 (5.9%)	0.699
Major hepatectomy	24 (1.6%)	8 (4.9%)	14 (2.7%)	25 (3.2%)	47 (3.2%)	0.392
Robotically assisted liver surgery	0 (0%)	8 (4.9%)	7 (1.4%)	311 (38.4%)	326 (21.9%)	<0.001
**Difficulty score**	112 (7.5%)					<0.001
IMM1		78 (50.0%)	300 (64.9%)	503 (66.2%)	881 (63.9%)	
IMM2		15 (9.6%)	33 (7.1%)	85 (11.2%)	133 (9.7%)	
IMM3		63 (40.4%)	129 (27.9%)	172 (22.6%)	364 (26.4%)	
Blood loss (ml), median (i.q.r.)	58 (3.9)	300 (100–600)	200 (50–400)	150 (50–300)	200 (50–400)	<0.001†
**Complications**	119 (8.0%)					0.031
None		121 (81.8%)	342 (71.5%)	581 (78.0%)	1044 (76.1%)	
Minor		14 (9.5%)	82 (17.2%)	104 (14.0%)	200 (14.6%)	
Major		13 (8.8%)	54 (11.3%)	60 (8.1%)	127 (9.3%)	
30-day mortality	1 (0.1%)	2 (1.2%)	2 (1.2%)	1 (0.2%)	3 (0.4%)	0.192
90-day mortality	1 (0.1%)	4 (2.4%)	7 (1.4%)	10 (1.2%)	21 (1.4%)	0.487
PS fully recovered at 30 days	304 (27.1%)	62 (76.5%)	330 (74.7%)	428 (76.0%)	820 (75.5%)	0.861

Values are *n* (%) unless otherwise stated. MILS, minimally invasive liver surgery; i.q.r., interquartile range; ASA, American Society of Anesthesiologists; PS, performance status according to Eastern Cooperative Oncology Group (ECOG); IMM, Institut Mutualiste Montsouris. *χ^2^ test, except †Mann–Whitney *U* test.

**Table 3:** Analysis of converted procedures

	Missing	Non-converted procedures (*n* = 1316)	Converted procedures (*n* = 174)	P*
Age (years), median (i.q.r.)	0 (0)	66 (56–73)	68.5 (59–77)	0.002†
**Sex**	0 (0%)			0.002
Male		684 (52.0%)	112 (64.4%)	
Female		632 (48.0%)	62 (35.6%)	
ASA grade ≥ III	52 (3.5%)	333 (26.3%)	68 (39.8%)	<0.001
Preoperative PS, ECOG ≥ 2	131 (9.1%)	72 (6.0%)	6 (3.8%)	0.245
**Indication for liver surgery**	1 (0.1%)			0.174
Primary liver malignancy		247 (18.8%)	35 (20.2%)	
Gallbladder cancer		53 (4.0%)	13 (7.5%)	
Benign or uncertain		366 (27.8%)	47 (27.2%)	
Liver metastases		650 (49.4%)	78 (45.1%)	
Tumour size ≥ 3cm	197 (13.2%)	461 (39.8%)	66 (48.5%)	0.051
> 3 tumours	47 (3.2%)	19 (1.5%)	5 (2.9%)	0.179
**Type of resection**	110 (6.4%)			0.089
Anatomical		362 (29.9%)	57 (33.3%)	
Non-anatomical		760 (62.9%)	95 (55.6%)	
Both		87 (7.2%)	19 (11.1%)	
Bilobar resection	98 (6.6%)	208 (17.0%)	58 (33.9%)	<0.001
Simultaneous ablation	0 (0%)	37 (2.8%)	7 (4.0%)	0.375
Simultaneous other surgery	178 (11.9%)	66 (5.8%)	11 (6.4%)	0.737
Major hepatectomy	24 (1.6%)	34 (2.6%)	13 (7.6%)	<0.001
**Difficulty score**	112 (7.5%)			<0.001
IMM1		79 (65.5%)	91 (53.2%)	
IMM2		104 (8.6%)	29 (17.0%)	
IMM3		313 (25.9%)	51 (29.8%)	
Robotically assisted liver surgery	0 (0%)	284 (21.6%)	42 (24.1%)	0.443
Blood loss (ml), median (i.q.r.)	58 (3.9)	150 (50–300)	350 (200–700)	<0.001†
**Complications**	119 (8.0%)			<0.001
None		939 (77.5%)	105 (65.6%)	
Minor complications		175 (14.5%)	25 (15.6%)	
Major complications		97 (8.0%)	30 (18.8%)	
30-day mortality	1 (0.1%)	3 (0.2%)	3 (1.7%)	0.003
90-day mortality	1 (0.1%)	15 (1.1%)	6 (3.4%)	0.015
PS fully recovered at 30 days	404 (27.1%)	736 (77.1%)	84 (64.1%)	<0.001
Resection margin ≥ 1 mm	519 (34.8%)	673 (78.2%)	85 (77.3%)	0.831

Values are *n* (%) unless otherwise stated. i.q.r., Interquartile range; ASA, American Society of Anesthesiologists; PS, performance status according to Eastern Cooperative Oncology Group (ECOG); IMM, Institut Mutualiste Montsouris. *χ^2^ test, except †Mann–Whitney *U* test.

Patients having RALS had higher ASA grades than those undergoing laparoscopic surgery (121 (38.8%) *versus* 280 (24.9%); *P* < 0.001), and were more likely to have gallbladder cancer (36 (11.0%) *versus* 30 (2.6%); *P* < 0.001), or a benign or uncertain indication (115 (35.3%) *versus* 298 (25.6%); *P* < 0.001), or bilobar resections (96 (30.8%) *versus* 170 (15.7%); *P* < 0.001) (*Table S2*). The groups were otherwise similar in terms of tumour characteristics and technical complexity. RALS was associated with a higher rate of PS recovery in 30 days (180 (80.7%) *versus* 640 (74.2%); *P* = 0.042) and less blood loss (100 (50–300) *versus* 200 (50–400) ml; *P* < 0.001).

### CRLM cohort

A total of 4488 patients underwent a first resection for CRLM, of whom 572 (12.7%) had MILS (*Table S3*). In the unmatched CRLM cohort, distributions were similar to those seen in the whole cohort, with more and larger metastases, more major resections, more bilobar operations, and more difficult resections in the OLS group. PSM resulted in a balanced cohort with 1060 patients, 530 in each group. In the matched cohort, patients undergoing MILS had less blood loss than the OLS cohort (200 (100–400) *versus* 300 (150–650) ml; *P* < 0.001) (*Table 4*). The radical resection rate was similar for MILS and OLS. The complication rate was lower in the MILS cohort (111 (23.2%) *versus* 148 (30.8%); *P* = 0.031), but the rate of major complications alone was not significantly different (*P* = 0.254). The 30- and 90-day mortality rates were similar in the two groups. LOS (analysed only from 2020) for MILS was shorter than that for OLS (4 (2.5–7) *versus* 6 (5–11) days; *P* = 0.009).

**Table 4:** Short-term outcomes after first liver resection for propensity score-matched colorectal cancer metastasis cohort

	Missing data	OLS (*n* = 530)	MILS (*n* = 530)	*P*†
Blood loss (ml), median (i.q.r.)	18 (1.7)	300 (150–650)	200 (100–400)	<0.001‡
Complications, any	101 (9.5%)	148 (30.8%)	111 (23.2%)	0.031
Major complications	101 (9.5%)	56 (11.6%)	43 (9.0%)	0.254
30-day mortality	2 (0.2%)	1 (0.2%)	1 (0.2%)	0.999
90-day mortality	2 (0.2%)	2 (0.4%)	6 (1.1%)	0.289
PS fully recovered at 30 days	333 (31%)	243 (70.6%)	284 (74.2%)	0.329
Resection margin ≥ 1 mm	208 (20%)	314 (75.3%)	339 (78.1%)	0.332
Length of hospital stay (days), median (i.q.r.)*	68 (24%)	6 (5–11%)	4 (2.5–7%)	0.009‡

Values are *n* (%) unless otherwise stated. *Analysed only using data from 2020 to 2023 (1133 in full cohort, 314 in matched cohort). OLS, open liver surgery; MILS, minimally invasive liver surgery; i.q.r., interquartile range; PS performance status according to Eastern Cooperative Oncology Group. †McNemar’s χ^2^ test, except ‡Wilcoxon signed-rank test.

**Fig. 3:**
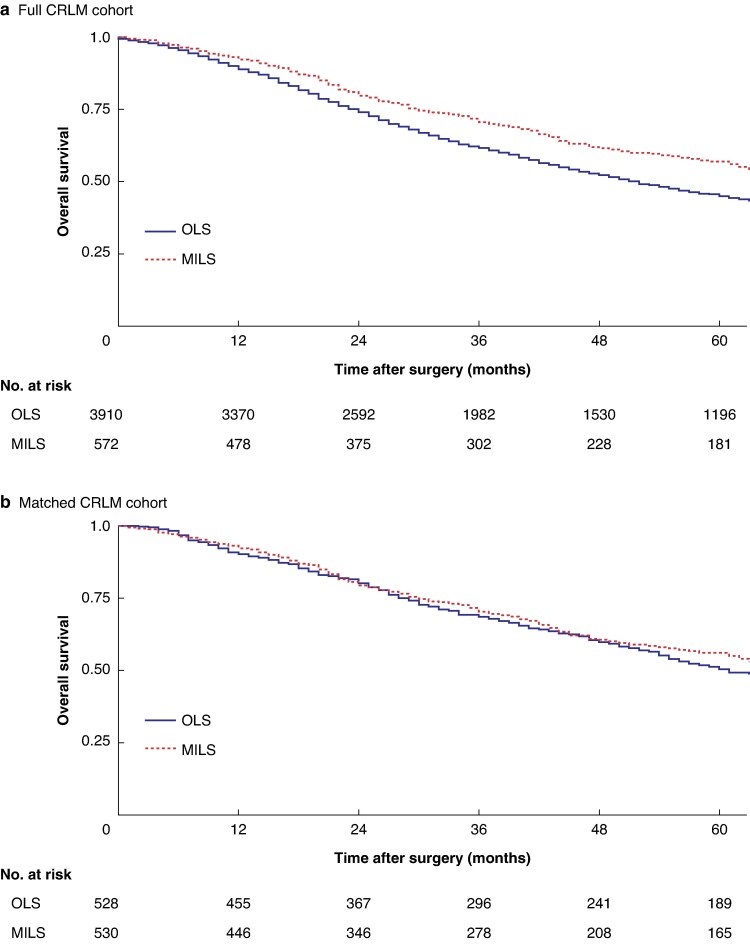
Kaplan–Meier overall survival curves for full CRLM and matched CRLM cohorts undergoing first liver resection stratified by surgical approach **a** Full colorectal liver metastasis (CLRM) cohort and **b** matched CRLM cohort. OLS, open liver surgery; MILS, minimally invasive liver surgery.

OS was 78 (63–93) months for MILS and 52 (49–55) months for OLS in the original, unmatched CRLM cohort (*P* < 0.001), and 78 (62–94) and 68 (54–82) months respectively in the matched cohort (*P* = 0.579) (*Fig. 3*). Cox regression analysis for the full CRLM cohort, adjusted for all variables used in PSM, confirmed that OS was similar after OLS and MILS (hazard ratio 0.95, 95% confidence interval 0.82 to 1.09; *P* = 0.448) (*Table S4*).

## Discussion

This national cohort study of the first 15 years of MILS in Sweden showed that MILS has been implemented safely without adversely effecting morbidity, mortality or OS, and has led to superior short-term outcomes. MILS became more frequent over time, and its implementation more tailored to the technical difficulties of the method. In the full cohort, MILS resulted in better short-term outcomes, with less bleeding, fewer major complications, and a lower 30-day mortality rate compared with OLS. These superior outcomes may have resulted from patient selection, as complex procedures and advanced tumour features were chosen for OLS.

In the early period, the rate of MILS was low in Sweden at 6.5%, and a high rate of resections with a high level of difficulty suggests an unawareness of the technical limitations of MILS at that time. However, rates of complications and mortality remained well within those later reported in trials such as the Oslo-CoMet study^4^ and the ORANGE II PLUS trial^6^. MILS was increasingly adopted in Sweden, and the 22% MILS rate in the late time period of this study is higher than the 12% reported from Poland in 2024^28^, but lower than the 25% in Japan in 2021^29^, and the 35% in France in 2024^30^. The rate of bilobar resections increased during the later two periods, whereas the IMM score and rate of anatomical resections decreased. International consensus meetings, guidelines, and difficulty scores likely contributed to a more tailored expansion of MILS during these periods^2,7,10–12^.

In the CRLM subgroup, PSM was used to compensate for selection bias in the surgical approach. In the matched cohort, MILS was associated with less bleeding and fewer complications, consistent with previous literature^2,4,6,7,9,20,31^. The radical resection rate was similar between MILS and OLS (78 *versus* 75%; *P* = 0.332) as was OS, in line with previous studies and reviews^4,6,32^. As radical margins are important for OS after resection of CRLMs, even in patients receiving modern chemotherapy, these results provide evidence of the oncological safety of MILS^33^. Despite the well balanced PSM, the improved outcomes in MILS could in part have resulted from residual confounding factors that could not be accounted for in the matching, such as detailed anatomical location, subtle differences in tumour biology, primary tumour status (tumour (T) and node (N) categories), and surgical experience, as these factors were not available in the registry data.

Progression along the learning curve for MILS requires many procedures to achieve proficiency^2,7,9,34,35^. A recent systematic review by Kuemmerli *et al*.^25^, which included studies both analysing institutional and individual surgeon’s learning curves, suggested that there are three standardized phases in analyses of the learning curve: competency, proficiency, and mastery, reached after 34, 50, and 58 procedures respectively. In the present study, the learning curve could be analysed only at institutional and national levels, and centres achieved the phases at various times, resulting in mixed outcomes. IMM3 resections and major resections decreased over time for both OLS and MILS, probably reflecting an adoption of parenchyma-sparing surgery, and altered patient selection criteria adapting to difficulties and limitations of MILS^2,7,11,36^. The 18% conversion rate in the late period is high compared with other published rates^4–6,8,28^. As expected, more complex operations were found among converted procedures, and worse outcomes. Nevertheless, short-term outcomes were similar to, or better, after MILS than OLS, in the full cohorts and the matched CRLM cohort, despite including converted procedures with worse outcomes in the MILS group in analyses.

RALS constituted 38% of MILS in the late period, and the data so far have demonstrated rapid and safe implementation of this method. Recently, Sijberden *et al*.^18^ compared RALS with laparoscopic liver surgery, using PSM, and reported fewer complications and conversions in the RALS group, as well as better intraoperative outcomes, and a higher rate of radical resections.

A strength of the study was presenting comprehensive national data, from a registry with high coverage. Limitations include the retrospective design, variability among centres in learning curves, and the registry’s lack of certain data or variables relevant to analyses, such as body mass index, T and N status of the primary tumour in patients with liver metastases, duration of operation, LOS being available only since 2020, and data on individual surgeon’s experience.

In conclusion, this study demonstrated an increasing and safe national adoption of MILS over time, including a rapid rise in RALS in the late period. MILS maintained or improved favourable short-term outcomes over time. This study provided evidence for safe implementation of MILS in Sweden without adverse effects on morbidity or mortality, and long-term survival after resection of CRLMs was unaffected.

## Supplementary Material

zraf164_Supplementary_Data

## Data Availability

Data are available through SweLiv following appropriate approval.
