# Supplementary material for: Nationwide implementation of minimally invasive liver surgery: population-based analysis
Source: BJS Open. 2026 Jan 21;10(1):zraf164. doi: 10.1093/bjsopen/zraf164 (PMC12822779; doi:10.1093/bjsopen/zraf164)
Supplement: zraf164_Supplementary_Data [file zraf164_supplementary_data.docx]

**Title**: **Nationwide implementation of minimally invasive liver surgery: a population-based analysis**

Authors: Emil Östrand (MD) ^1,2^, Jenny Rystedt (MD, PhD) ^1,2^, Bobby Tingstedt (MD, PhD) ^1,2^, Bodil Andersson (MD, PhD) ^1,2^

^1^ Department of Clinical Sciences Lund, Surgery, Lund University, Sweden

^2^ Department of Surgery, Skåne University Hospital, Lund, Sweden

**Corresponding author.** Emil Östrand, MD, Department of Surgery, Skåne University Hospital Lund, SE-221 85 Lund, Sweden. Phone: +46 46 172383. E-mail: [emil.ostrand@med.lu.se](mailto:emil.ostrand@med.lu.se) **ORCID ID 0000-0001-7621-9541**;

**Supplementary Materials - Index**

| **Supplementary methods** |  |
| --- | --- |
| Appendix S1 | *Page 2* |
| **Supplementary Figures and Tables** |  |
| Table S1 | *page 3* |
| Table S2 | *page 4* |
| Table S3 | *page 5* |
| Table S4 | *page 6* |
| **References** | *page 7* |

**Supplementary methods**

**Appendix S1. Difficulty grading according to Institut Mutualiste Montsouris**

Difficulty was graded according to Institut Mutualiste Montsouris (IMM). In IMM group 1, wedge resections and left lateral sectionectomies were included; in group 2, anterolateral segmentectomies and left hepatectomies were included; and in group 3, posterosuperior segmentectomies, right posterior sectionectomies, right anterior sectionectomies, right hepatectomies, central hepatectomies, and extended left or right hepatectomies were included. ^1^

**Supplementary Figures and Tables**

Table S1. Proportion of MILS over time

| **Indication for liver surgery** | | **OLS** | **MILS** | **p-value** |
| --- | --- | --- | --- | --- |
|  | |  |  |  |
| **Primary liver malignancy** | |  |  | **<0.001** |
|  | Early, 2009-2013 | 374 (94) | 24 (6.0) |  |
|  | Middle, 2014-2018 | 621 (88) | 825 (12) |  |
|  | Late, 2019-2023 | 622 (78) | 176 (22) |  |
| **Gallbladder cancer** | |  |  | **<0.001** |
|  | Early, 2009-2013 | 128 (97) | 4 (3.0) |  |
|  | Middle, 2014-2018 | 210 (97) | 7 (3.2) |  |
|  | Late, 2019-2023 | 210 (79) | 55 (21) |  |
| **Benign, or uncertain** | |  |  | **<0.001** |
|  | Early, 2009-2013 | 237 (87) | 36 (13) |  |
|  | Middle, 2014-2018 | 353 (73) | 133 (27) |  |
|  | Late, 2019-2023 | 348 (59) | 244 (41) |  |
| **Liver metastases** | |  |  | **<0.001** |
|  | Early, 2009-2013 | 1636 (94) | 100 (5.8) |  |
|  | Middle, 2014-2018 | 2005 (87) | 294 (13) |  |
|  | Late, 2019-2023 | 1733 (84) | 334 (16) |  |
|  | |  |  |  |
|  | | | | |

**Table S1.** Values are numbers (percent). Variables compared using Chi-Square test. OLS=Open liver surgery. MILS=Minimally invasive liver surgery. n=9977. Missing data = 4 (0.04%).

Table S2. Robotic assisted liver surgery vs. laparoscopic liver surgery

| **Variable** | | **Missing**  n (%) | **Laparoscopic** n=1164 | **RALS** n=321 | **p-value** |
| --- | --- | --- | --- | --- | --- |
|  | |  |  |  |  |
| **Age in years, median (IQR)** | | 2 (0.02) | 66 (57-73) | 67 (55-76) | 0.096 |
| **Gender, male** | | 0 | 627 (54%) | 169 (52%) | 0.517 |
| **ASA-score ≥3** | | 52 (3.5) | 280 (25%) | 121 (39%) | **<0.001** |
| **PS pre-op, ECOG ≥2** | | 136 (9.1) | 63 (6.0%) | 15 (5.1%) | 0.563 |
| **Indication for liver surgery** | | 1 (0.07) |  |  | **<0.001** |
|  | Primary liver malignancy |  | 217 (19%) | 65 (20%) |  |
|  | Gallbladder cancer |  | 30 (2.6%) | 36 (11%) |  |
|  | Benign. or uncertain |  | 298 (26%) | 115 (35%) |  |
|  | Liver metastases |  | 618 (53%) | 110 (34%) |  |
| **Tumour size ≥3cm** | | 197 (13) | 417 (40%) | 110 (44%) | 0.200 |
| **Number of tumours >3** | | 47 (3.2) | 20 (1.8%) | 4 (1.3%) | 0.527 |
| **Type of resection** | | 110 (7.4) |  |  | 0.157 |
|  | Anatomical resection |  | 338 (32%) | 81 (26%) |  |
|  | Non-anatomical |  | 649 (61%) | 206 (66%) |  |
|  | Both |  | 81 (7.6%) | 25 (8.0%) |  |
| **Bi-lobar resection** | | 98 (6.6) | 170 (16%) | 96 (31%) | **<0.001** |
| **Simultaneous ablation** | | 0 | 36 (3.1%) | 8 (2.5%) | 0.547 |
| **Simultaneous other surgery** | | 178 (12) | 57 (5.7%) | 20 (6.6%) | 0.559 |
| **Major hepatectomy** | | 24 (1.6) | 36 (3.1%) | 11 (3.5%) | 0.736 |
| **Difficulty** | | 112 (7.5) |  |  | 0.148 |
|  | IMM1 |  | 678 (64%) | 203 (65%) |  |
|  | IMM2 |  | 96 (9.0%) | 37 (12%) |  |
|  | IMM3 |  | 292 (27%) | 72 (23%) |  |
| **Bleeding in ml, median (IQR)** | | 58 (3.9) | 200 (50-400) | 100 (50-300) | **<0.001** |
| **Complications** | | 119 (8.0) |  |  | 0.784 |
|  | None |  | 811 (76%) | 233 (77%) |  |
|  | Minor complications |  | 157 (15%) | 43 (14%) |  |
|  | Major complications |  | 102 (9.5%) | 25 (8.3%) |  |
| **Mortality 30 days** | | 1 (0.07) | 5 (0.4%) | 1 (0.3%) | 0.756 |
| **Mortality 90 days** | | 1 (0.07) | 17 (1.5%) | 4 (1.2%) | 0.751 |
| **PS fully recovered at 30 days** | | 404 (27) | 640 (74%) | 180 (81%) | **0.042** |
| **Length of stay*, median days (IQR)** | | 98 (16) | 4 (2-6) | 3 (2-7) | 0.755 |
| **Conversions to open** | | 0 | 132 (11) | 42 (13) | 0.443 |
|  | |  |  |  |  |
|  | |  |  |  |  |

Table S2. Values are numbers (percent) unless otherwise noted. RALS = Robotic assisted liver surgery. ASA= American Society of Anaesthesiologists, IQR = Inter Quartile Range. IMM = Institut Mutualiste Montsouris. PS= Performance status, ECOG=Eastern Cooperative Oncology Group. *=analysed only in data from 2020-2023, n=629. Distributions compared using Chi-Square for categorical data and Kruskal-Wallis-test for continuous data.

**Table S3. Colorectal Cancer Metastases cohorts, matched variables**

|  | | | **Full cohort** | | | | | **Propensity Score Matched cohort** | | | | |
| --- | --- | --- | --- | --- | --- | --- | --- | --- | --- | --- | --- | --- |
| **Variable** | | | **Missing data** n (%) | **OLS** n=3916 | **MILS** n=572 | **SMD** | **p-value** | **OLS**   n=530 | **MILS** n=530 | **SMD** | **p-value** |  |
|  | | |  |  |  |  |  |  |  |  |  |  |
| *Matched variables* | | |  |  |  |  |  |  |  |  |  |  |
| **Age in years (IQR)** | | | 2 (0.0) | 67 (59-73) | 68 (71-75) | 0.08 | 0.085 | 69 (62-74) | 68 (61-75) | 0.05 | 0.376 |  |
| **Gender, male** | | | 0 | 2455 (63) | 348 (61) | 0.04 | 0.393 | 338 (64) | 331 (62) | 0.03 | 0.656 |  |
| **ASA-score ≥3** | | | 37 (0.8) | 1014 (26) | 139 (24) | 0.04 | 0.404 | 119 (22) | 132 (25) | 0.06 | 0.348 |  |
| **Extrahepatic metastases** | | | 12 (0.3) | 443 (11) | 53 (9.3) | 0.07 | 0.147 | 41 (7.7) | 51 (9.6) | 0.07 | 0.275 |  |
| **Largest metastasis ≥3cm** | | | 57 (1.3) | 1581 (40) | 195 (34) | 0.14 | **0.002** | 183 (35) | 186 (35) | 0.01 | 0.851 |  |
| **>3 liver metastases** | | | 39 (0.9) | 912 (24) | 14 (2.5) | 0.66 | **<0.001** | 15 (2.8) | 14 (2.6) | 0.01 | 0.851 |  |
| **Type of resection** | | | 22 (0.5) |  |  | 0.55 | **<0.001** |  |  | 0.09 | 0.362 |  |
|  | Anatomical | |  | 1280 (33) | 155 (28) |  |  | 129 (24) | 149 (28) |  |  |  |
|  | Non-anatomical | |  | 1482 (38) | 337 (61) |  |  | 337 (64) | 323 (61) |  |  |  |
|  | Both | |  | 1151 (29) | 61 (11) |  |  | 64 (12) | 58 (10) |  |  |  |
| **Bi-lobar resections** | | | 60 (1.3) | 1757 (45) | 102 (18) | 0.62 | **<0.001** | 106 (20) | 95 (18) | 0.05 | 0.398 |  |
| **Simultaneous ablation** | | | 0 | 559 (14) | 26 (4.5) | 0.34 | **<0.001** | 20 (3.8) | 25 (4.7) | 0.05 | 0.446 |  |
| **Major hepatectomy** | | | 0 | 1443 (37) | 16 (2.8) | 0.94 | **<0.001** | 17 (3.2) | 16 (3.0) | 0.01 | 0.860 |  |
| **Difficulty** | | | 36 (0.8) |  |  | 0.65 | **<0.001** |  |  | 0.06 | 0.655 |  |
|  | | IMM1 |  | 1173 (30) | 329 (60) |  |  | 301 (57) | 312 (59) |  |  |  |
|  | | IMM2 |  | 321 (8.2) | 48 (8.7) |  |  | 44 (8.3) | 47 (8.9) |  |  |  |
|  | | IMM3 |  | 2406 (62) | 175 (32) |  |  | 185 (35) | 171 (32) |  |  |  |
| **Neoadjuvant chemotherapy** | | | 61 (1.4) | 1178 (30) | 78 (14) | 0.41 | **<0.001** | 644 (12) | 75 (14) | 0.06 | 0.317 |  |
|  | | |  |  |  |  |  |  |  |  |  |  |

**Table S3**. Balance of characteristics of patients having a first resection for colorectal liver metastases, in full cohort of CRLM, and the matched cohort. Values are numbers (percent) unless otherwise noted. Variables compared using Chi-Square for categorical data and Kruskal-Wallis-test for continuous data in full cohort and Wilcoxon signed rank test for continuous data and McNemar’s Chi-Squared test for categorical data in the matched cohort. SMD= Standardized mean difference. OLS=Open liver surgery. MILS=Minimally invasive liver surgery. ASA= American Society of Anaesthesiologists. IQR = Inter Quartile Range. Propensity score matching was performed with 1:1 matching using nearest neighbour matching with a caliper width of 0.01.

**Table S4. Cox regression with shared frailty in colorectal liver metastases cohorts**

|  | | **Matched cohort** | | **Full cohort** | |
| --- | --- | --- | --- | --- | --- |
| **Variable** | | **HR (95% CI)** | **p-value** | **HR (95% CI)** | **p-value** |
| **Surgical approach** | |  | 0.999 |  | 0.448 |
|  | OLS | 1 (Ref.) |  | 1 (Ref.) |  |
|  | MILS | 0.9 (0.7 - 1.1) |  | 0.9 (0.8 - 1.1) |  |
|  | | | | | |

**Table S4.** Cox regression with shared frailty for matched cohort and Cox regression adjusted for variables in propensity score for full cohort. Hazard ratios can be interpreted in the same way as a “regular” Cox regression, the frailty term is to account for the within match dependence. OLS=Open Liver surgery. MILS=Minimally invasive liver surgery.

# References

1. Kawaguchi Y, Fuks D, Kokudo N and Gayet B. Difficulty of laparoscopic liver resection: Proposal for a new classification. *Ann Surg*. 2018; **267**: 13-17.
